# Electron Spin Broken‐Symmetry of Fe–Co Diatomic Pairs to Promote Kinetics of Bifunctional Oxygen Electrocatalysis for Zinc–Air Batteries

**DOI:** 10.1002/advs.202401187

**Published:** 2024-06-14

**Authors:** Xiaokang Li, Jian Qin, Qingxin Lin, Xiaoyu Yi, Cheng Yan, Jianhua Zhang, Jinjuan Dong, Kang Yu, Shenglong Zhang, Chong Xie, Huijuan Yang, Wei Xiao, Wenbin Li, Jingjing Wang, Xifei Li

**Affiliations:** ^1^ Institute of Advanced Electrochemical Energy and School of Materials Science and Engineering Xi'an University of Technology Xi'an Shaanxi 710048 China; ^2^ Shaanxi International Joint Research Center of Surface Technology for Energy Storage Materials Xi'an Shaanxi 710048 China; ^3^ Department of Materials Science and Engineering Macau University of Science and Technology Macau 999078 China

**Keywords:** Fe–Co bimetallic, flexible wearables, single‐atom catalysts, unsymmetric configuration, Zinc–air batteries

## Abstract

Designing bifunctional catalysts to reduce the oxygen evolution reaction (OER) and oxygen reduction reaction (ORR) reaction barriers while accelerating the reaction kinetics is perceived to be a promising strategy to improve the performance of Zinc–air batteries. Unsymmetric configuration in single‐atom catalysts has attracted attention due to its unique advantages in regulating electron orbitals. In this work, a seesaw effect in unsymmetric Fe–Co bimetallic monoatomic configurations is proposed, which can effectively improve the OER/ORR bifunctional activity of the catalyst. Compared with the symmetrical model of Fe–Co, a strong charge polarization between Co and Fe atoms in the unsymmetric model is detected, in whom the spin‐down electrons around Co atoms are much higher than those spin‐up electrons. The seesaw effect occurred between Co atoms and Fe atoms, resulting in a negative shift of the d‐band center, which means that the adsorption of oxygen intermediates is weakened and more conducive to their dissociation. The optimized reaction kinetics of the catalyst leads to excellent performance in ZABs, with a peak power density of 215 mW cm^−2^ and stable cycling for >1300 h and >4000 cycles. Flexible Zinc–air batteries have also gained excellent performance to demonstrate their potential in the field of flexible wearables.

## Graphical abstract

The electronic structure of catalysts plays an important role in their catalytic performance for Zinc–air batteries. An unsymmetric configuration between Fe–Co single atoms is designed in this work and a seesaw effect is proposed, which can enhance the delocalization of d‐band electrons and form spin polarization effects, thereby improving the overall electrocatalytic activity.

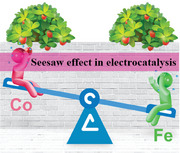

## 1 Introduction

As global energy demand continues to increase, it is urgent to develop more advanced energy storage equipment. Zinc–air batteries (ZABs) are sustainable, efficient, and clean energy devices, that have been widely concerned in recent years.^[^ ^1^ ^]^ The main challenge of Zinc–air batteries is concentrated on the air cathode, where a large overpotential is required to drive the slow oxygen reduction reaction (ORR)/oxygen evolution reaction (OER) in kinetics during discharging/charging, accompanied by polarization.^[^ ^2^ ^]^ Electrocatalysts with ORR/OER dual function are currently the focus of research.^[^ ^3^ ^]^

Both ORR and OER involve multiphase reactions, including the formation and conversion of intermediates such as OOH^*^, O^*^, and OH^*^, the core of which lies in the process of “adsorption‐conversion‐desorption”.^[^ ^4^ ^]^ Single atom catalysts (SACs) have received great attention in this multiphase catalytic reaction due to their high atomic utilization rate, and have been able to replace commercially Pt‐based and Ir/Ru‐based catalysts.^[^ ^5^ ^]^ Numerous theoretical and experimental explorations have shown that an isolated single metal‐N‐C (M‐N‐C) structure can serve as an ideal bifunctional catalyst.^[^ ^6^ ^]^ Among them, the single metal‐N_4_ configuration with a symmetric four‐coordinated planar structure is first considered the most effective catalytic site in M‐N‐C, which is especially supported by theoretical calculation and experiment.^[^ ^7^ ^]^ However, it is well known that the decision step is the most important in multi‐step reactions.^[^ ^8^ ^]^ In symmetric configurations, the isotropic electron distribution does not show catalytic differences between different intermediate reactions. In addition, the large electronegativity of symmetric adjacent nitrogen atoms around the metal site in M‐N_4_ leads to inappropriate free energy of intermediate product adsorption.^[^ ^9^ ^]^

The electronic structure of catalysts plays a crucial role in catalytic reactions.^[^ ^10^ ^]^ Common descriptors such as d‐band center, e.g., electron filling, and spin polarization can be used to predict catalytic activity from the “adsorption–conversion–desorption” perspective.^[^ ^11^ ^]^ For example, the interaction between the d‐orbitals of metal atoms causes charge transfer, which affects the catalytic performance of the catalysts.^[^ ^12^ ^]^ Various methods such as doping, hetero‐interface effect, and defect introduction were used to design specific charge distribution to improve its catalytic activity.^[^ ^13^ ^]^ However, the electronic structure of the partially occupied 3d‐orbitals of central metal atoms is inseparable from their coordination geometry.^[^ ^14^ ^]^ The design of unsymmetric structures is another effective approach.^[^ ^15^ ^]^ Unsymmetric electron and molecular configurations can be formed between atoms with different electron spin densities, atomic radii, and electronegativity, which can enhance the delocalization of d‐band or change the charge density difference to varying degrees, thus improving the electrocatalytic activity of SACs.^[^ ^16^ ^]^ In addition, finite spin polarization can create a stray field that traps O_2_ molecules and intermediates and keeps the spin preserved, but it must be mild to avoid strong adsorption.^[^ ^17^ ^]^

Herein, a simple and effective approach is adopted to design unsymmetric bimetallic single‐atom catalysts between Fe and Co. Molten NaCl is used to provide an ionic liquid environment, promoting the complex of Fe/Co with N atoms to form C─N_3_─Fe─Co─N_4_─C structure. The unsymmetrical chemical environment and coordination structure of the bimetal site were determined. The effects of symmetry break between active sites on their intrinsic activities were discussed in depth. The combination of experiments and theoretical calculations has demonstrated the lifting effect of Fe sites on Co sites in unsymmetric structures, forming a seesaw effect to reduce the energy barrier of the decisive step in multiphase catalytic reactions and improve the bifunctional catalytic performance of the material. The seesaw effect proposed in this study will provide a new idea for designing bifunctional catalysts with excellent performance.

## 2 Results and Discussion

### 2.1 Synthesis and Characterization of Catalysts

**Figure 1:**
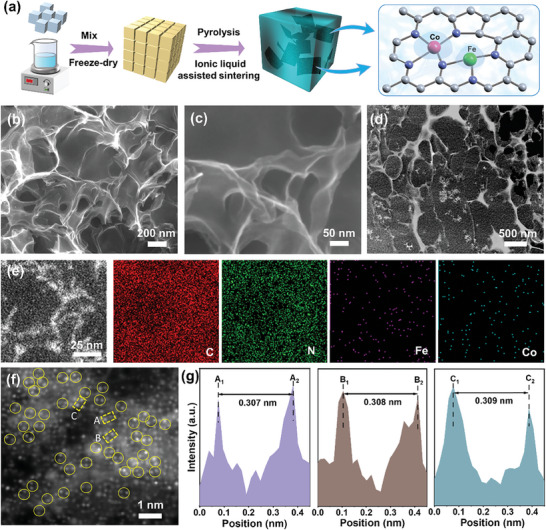
a) Schematic illustration of the fabrication of Fe–Co(DSA)@3DNC. b,c) SEM, d) TEM, and e) STEM images and elemental mapping of the Fe–Co(DSA)@3DNC. f) HAADF‐STEM image of Fe–Co(DSA)@3DNC. Isolated atoms are highlighted in yellow circles. g) Interatomic distance analysis based on f).

The synthesis of Fe–Co dual single‐atom catalyst anchored with 3D nitrogen‐doped carbon (Fe–Co(DSA)@3DNC) is shown in **Figure** **1**a. NaCl was used as a template first. Ammonium citrate, melamine, and metal sources were dissolved in deionized water, and a homogenized solution was obtained after full stirring. Next, the resulting solution was placed at a low temperature to ensure that it freezes completely into ice, and then subjected to freeze drying. In this case, NaCl crystallizes and forms regular nanocrystal arrays.^[^ ^18^ ^]^ Meanwhile, carbon, nitrogen, and metal sources are uniformly dispersed on the surface of the NaCl template. Due to the effect of NaCl crystallization force and the rich surface it provides, the thickness of the two‐dimensional mixture will be limited to the nanometer scale.^[^ ^19^ ^]^ Thirdly, the precursor was placed in a tubular furnace and held at 1000 °C for 2 h in an argon atmosphere. At this time, the molten NaCl (melting point of 801 °C) acts as an ionic liquid that not only limits the thickness of the carbon sheet but also assists the complex of Fe/Co with N atoms to form C–N_x_–Fe–Co–N_x_–C structure. Finally, the NaCl template was repeatedly cleaned with deionized water to obtain the catalyst named Fe–Co(DSA)@3DNC. For comparison, Fe and Co nonmetallic catalysts were prepared by the same method, denoted as Fe(SA)@3DNC and Co(SA)@3DNC.

Scanning electron microscopy (SEM, Figure 1b,c) and transmission electron microscopy (TEM, Figure 1d) images show that the Fe–Co(DSA)@3DNC catalyst is composed of ultra‐thin carbon networks with a porous structure which can promote gas and electron transport in the reaction. No nanoparticles are observed. The corresponding elemental mapping images (Figure 1e) clearly show the uniform distribution of Fe and Co elements, which proves the monatomic structure. Furthermore, the high‐angle annular darkfield STEM (HAADF‐STEM) image in Figure 1f confirms the atomic configuration of Fe and Co in the Fe–Co(DSA)@3DNC catalyst, and no atomic clusters or nanocrystals are observed (The image has been filtered, Figure S1 (Supporting Information) is the image before filtering). It shows that the high‐temperature treatment of 1000 °C does not agglomerate the metal atoms into nanocrystals, but realizes isolated single atoms anchored 3DNC network under the premise of maintaining high stability, which can be ascribed to the isolation effect of ionic liquids (molten NaCl) on atoms.^[^ ^20^ ^]^ Interestingly, it seems that these atoms tend to exist in pairs (highlighted by the yellow cycle), as shown in Figure 1g. The distances of atomic pairs are randomly measured, most of which are concentrated ≈0.307–0.308 nm, and the statistical results are shown in Figure S2 (Supporting Information).^[^ ^10^ ^]^ Besides, SEM images of Fe(SA)@3DNC and Co(SA)@3DNC show topography consistent with Fe–Co(DSA)@3DNC (Figure S3, Supporting Information).

**Figure 2:**
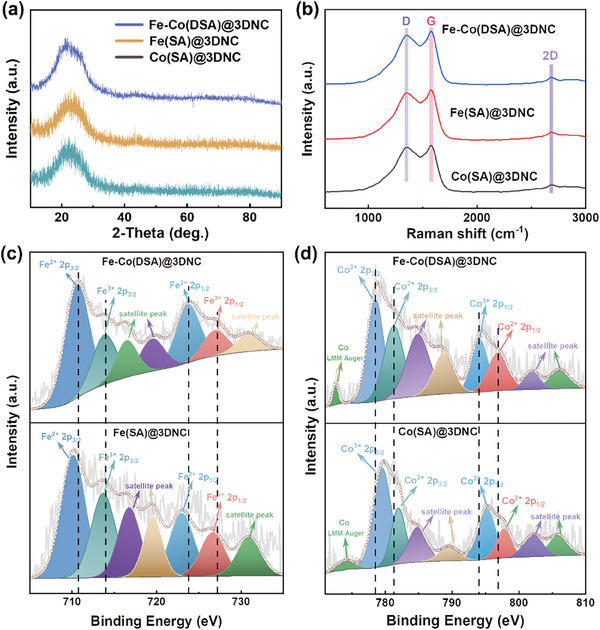
a) XRD, b) Raman, and c,d) High‐resolution XPS spectra of Fe–Co(DSA)@3DNC, Fe(SA)@3DNC and Co(SA)@3DNC.

**Figure** **2**a shows the XRD patterns of Fe(SA)@3DNC, Co(SA)@3DNC, and Fe–Co(DSA)@3DNC. It can be seen that all three curves have only one obvious peak, which represents graphitized carbon. A small peak at 43° belongs to (101) crystalographic plane of graphite, resulting from the catalysis of single atoms. No Fe or Co peaks are detected. Figure 2b shows the Raman results, where all three samples show peaks at 1350, 1580, and 2680 cm^−1^, corresponding to the D peak (disordered defect vibration), G peak (in‐plane telescopic vibration of carbon atoms), and 2D peak (diphoton resonance) of carbon materials, respectively. A strong D peak indicates abundant defects in the 3DNC which can promote the catalytic conversion process of oxygen.^[^ ^21^ ^]^ The I_D_/I_G_ of the three materials are ≈0.95, indicates that the defects and graphitization degrees of the three materials are basically the same. Except for carbon peaks, there are no other peaks. To further elucidate the surface chemistry and electronic structure of the samples, X‐ray photoelectron spectroscopy (XPS) characterization was performed. As shown in Figure S4 and Table S1 (Supporting Information), the characteristic peaks of C1s, N1s, and O1s appear in the same location in the three samples.^[^ ^22^ ^]^ The high‐resolution peaks of Fe and Co elements in Fe–Co(DSA)@3DNC, Fe(SA)@3DNC, and Co(SA)@3DNC are pinned and fitted, and the results are shown in Figure 2c,d. Fe and Co both exist in divalent and trivalent forms, which is related to their coordination with N. Significantly, the Fe peaks in Fe–Co(DSA)@3DNC are shifted to high energy compared with Fe(SA)@3DNC and the Co peaks in Fe–Co(DSA)@3DNC are shifted to low energy compared with Co(SA)@3DNC.^[^ ^23^ ^]^ This phenomenon indicates that there is electron transfer between Fe and Co atoms in Fe–Co(DSA)@3DNC, which may be related to the occurrence of diatomic pairs. In this case, Fe is in a state of losing electrons, and the p‐electron binding energy is higher, which is difficult to ionize; Co is in a state of obtaining electrons, and the p‐electron binding energy is low, which is easier to ionize. Therefore, it can be inferred that Co atoms strip and absorb electrons from Fe atoms, forming electron‐rich regions near Co atoms, which results in the transfer of delocalized electrons from Fe to Co atoms. The metal content was determined by inductively coupled plasma‐mass spectrometry (ICP‐MS). The elemental contents of Fe and Co in Fe–Co(DSA)@3DNC are 1.93% and 1.20%, respectively, while the compared samples have the same metallic content (Table S2, Supporting Information).

### 2.2 Identification and Establishment of Unsymmetric Coordination Configurations

**Figure 3:**
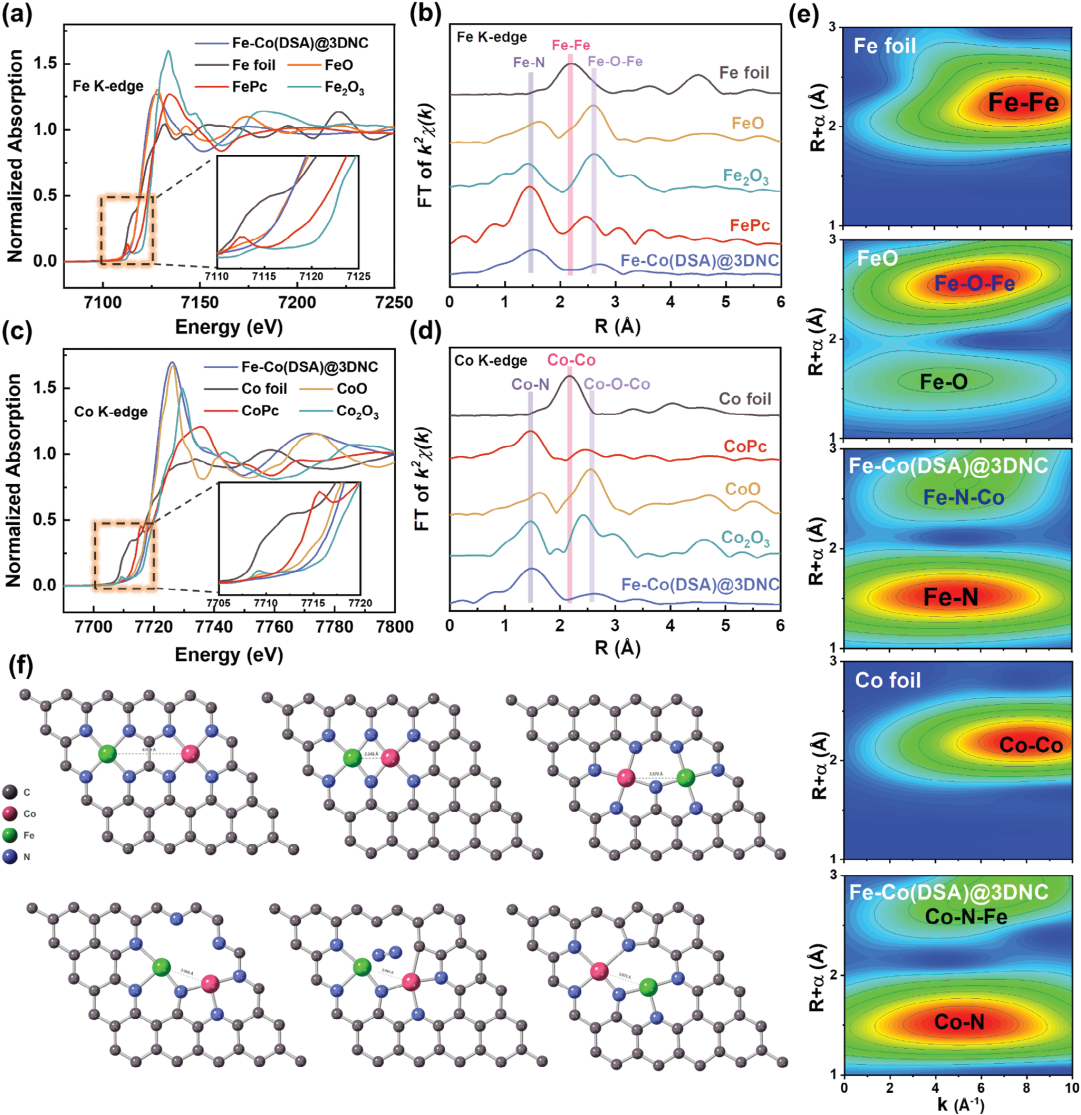
a,b) Fe K‐edge XANES and corresponding Fourier‐transform EXAFS spectra, c,d) Co K‐edge XANES and corresponding Fourier‐transform EXAFS spectra and e) Wavelet transform of the EXAFS data of Fe–Co(DSA)@3DNC and reference samples. f) Coordination model of Fe–Co bimetal with N/C, the last of which is consistent with the results of a–e).

X‐ray absorption fine structure (XAFS) was analyzed to study the electronic state and atomic properties of Fe and Co in Fe–Co(DSA)@3DNC catalyst.^[^ ^24^ ^]^ X‐ray absorption near edge structure (XANES) is shown in **Figure** **3**a,c, the higher energy curve represents a higher attraction to electrons.^[^ ^25^ ^]^ It can be seen that compared with FePc and CoPc, the Fe curve in Fe–Co(DSA)@3DNC catalyst moves onward lower energy and the Co curve moves oward higher energy, indicating electron transfer from Fe to Co, which is consistent with the XPS results. To confirm the local coordination environment of Fe and Co sites, the extended X‐ray absorption fine structure (EXAFS) curve of the Fourier transform in R space was further analyzed. The EXAFS curve of Fe in Fe–Co(DSA)@3DNC catalyst shows a strong peak at 1.5 Å, which is attributed to Fe–N coordination (FePc), while the EXAFS curve of Co shows a strong peak at 1.5 Å, which represents Co–N coordination (CoPc). By comparing various reference sample curves, no Fe–Fe and Co–Co signals are observed on the two EXAFS curves, but there is a slight perturbation near 3 Å. Wavelet transform (WT) is combined to reveal the local coordination environment of Fe and Co atoms in k and R space (Figure 3e).^[^ ^26^ ^]^ After analysis and comparison, Fe–Co(DSA)@3DNC catalyst has strong coordination absorption peaks of Fe–N, and Co–N, while the low‐intensity perturbation near 3 Å could be attributed to the coordination mode of Fe–N–Co formed by diatomic pairs model. Generally, the synchrotron radiation plot line is not very accurate, especially since the distance will have an error of ≈0.5 Å. Therefore, an elaborate structure model fitting is employed for further quantitative analysis, and the accurate coordination number and atomic spacing are obtained. Figures S5–S8 (Supporting Information) show the EXAFS fitting curves of Fe–Co(DSA)@3DNC catalysts in R space and k space. The detailed fitting parameters and results are shown in Tables S3 and S4 (Supporting Information). As can be seen in the fitted data, when used one path (Fe–N path or Co–N path) in the fitting of Fe K‐edge and Co K‐edge EXAFS analysis in R space, it cannot accurately demonstrate the coordination relationship of the second shell. After adding the second path (Fe–Co path), the second shell is accurately fitted, proving that Fe and Co are the second shell of each other, and the existence form is most likely Fe–N–Co. The final results show that the coordination between Fe and N atoms in Fe–Co(DSA)@3DNC catalyst is ≈3, while the coordination number between Co and N atoms is ≈4. The average atomic spacing is ≈3.14–3.19 Å, which is consistent with the observation of ACTEM in Figure 1f. Based on the above results, we consider six possible Fe–Co(DSA)@3DNC models composed of Fe, Co, N, and C atoms, as shown in Figure 3f. After density functional theory (DFT) theoretical calculation, the atomic structure is optimized. The coordination number and atomic spacing of Fe–N, Co–N, and Fe–Co are compared. The unsymmetric Fe–Co bimetallic structure model is confirmed in the Fe–Co(DSA)@3DNC (see yellow circle in Figure 3f), where Co coordinates with 4 N, Fe coordinates with 3 N, and the atomic spacing between Co–Fe is ≈3.073 Å.

### 2.3 Electrochemical Oxygen Reduction and Oxygen Evolution Performance

**Figure 4:**
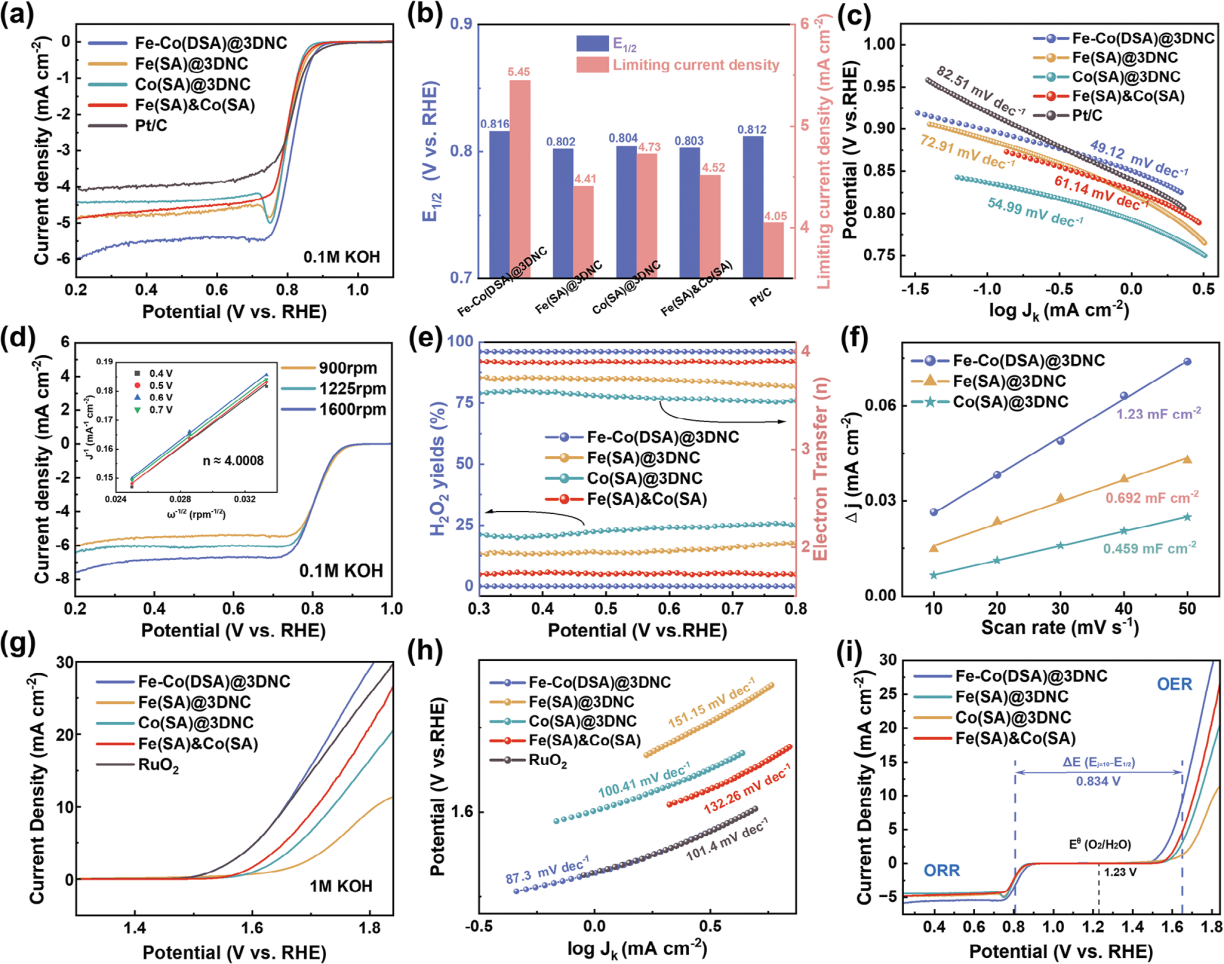
a) ORR linear sweep voltammetry curve, b) half‐wave potential and limit current density, and c) Tafel slope of the catalysts. d) Linear sweep voltammetry of Fe–Co(DSA)@3DNC at different speeds (inset electron transfer number curve after calculation of K–L equation). e) Hydrogen peroxide yield and electron transfer number plot tested at rotating ring disk electrode, f) calculated cdl value, g) OER linear sweep voltammetry curve, h) Tafel slope, and i) OER/ ORR bifunctional curve of the catalysts.

ORR and OER performance of Fe(SA)@3DNC, Co(SA)@3DNC, their mechanical mixture (Fe(SA)&Co(SA)), and precious metal Pt/C were first evaluated, as shown in **Figure** **4**.^[^ ^27^ ^]^ Among them, Fe–Co(DSA)@3DNC has the highest half‐wave potential (0.816 V) as well as the largest limit current density (5.45 mA cm^−2^) in ORR, which highlights the critical role of Fe–Co diatomic pair structures (see Figure 4a,b). The peaks in the LSV curves of Fe(SA)@3DNC and Co(SA)@3DNC are caused by the adsorption of a large amount of O_2_ in the early stage of the reaction, which cannot be reduced in time. Figure 4c shows the ORR Tafel slope of these catalysts, in which Fe–Co(DSA)@3DNC possess a small slope value of 49.13 mV dec^−1^, all less than Fe(SA)@3DNC (72.91 mV dec^−1^), Co(SA)@3DNC (54.99 mV dec^−1^), Fe(SA)&Co(SA) (61.14 mV dec^−1^), and Pt/C (82.51 mV dec^−1^). As shown in Figure S9 (Supporting Information), the CV of the three catalysts in the Ar‐saturated solution all exhibit rectangular curves with no peaks, corresponding to the double‐layer capacitance. In the O_2_‐saturated solution, reduction peaks appear in the CV curve, which are related to ORR. The starting potential and intensity of the cathodic peak in Fe–Co(DSA)@3DNC are relatively high, reflecting its smaller overpotential and larger current density. ORR performance of Fe–Co(DSA)@3DNC at different rotational speeds is shown in Figure 4d. The electron transfer number n is ≈4 calculated by the Koutecky–Levich (K–L) equation,^[^ ^28^ ^]^ which illustrates that Fe–Co(DSA)@3DNC can directly reduce oxygen through the four‐electron pathway at a relatively low overpotential. At the same time, the rotating ring disk electrode (RRDE) test was used to detect the peroxide (HO^2−^) yield and electron transfer number of each catalyst at different voltages, and the results are shown in Figure 4e. The peroxide yield of Fe–Co(DSA)@3DNC does not exceed 1%, and the corresponding electron transfer number is ≈3.98–3.99, while the peroxide yields of Fe(SA)@3DNC, Co(SA)@3DNC, and Fe(SA)&Co(SA) are all higher than 5%, and the electron transfer numbers are also >3.9, which proves that Fe–Co(DSA)@3DNC have high selectivity for oxygen reduction. In Figures 4f and S10 (Supporting Information), the electrochemically active surface area (ECSA) of Fe–Co(DSA)@3DNC is calculated using the electric double‐layer capacitance method (Cdl), and the Cdl value of Fe–Co(DSA)@3DNC (1.23 mF cm^−2^) is greater than that of Fe(SA)@3DNC (0.692 mF cm^−2^) and Co(SA)@3DNC (0.459 mF cm^−2^), which shows that Fe–Co(DSA)@3DNC has a high electrochemically active specific surface area. In addition, Fe–Co(DSA)@3DNC also exhibits excellent oxygen OER performance under alkaline conditions. Figure 4g,h shows the OER performance of these catalysts, among which Fe–Co(DSA)@3DNC has the smallest potential of 1.65 V at 10 mA cm^−2^ and smallest tafel slope of 87.30 mV dec^−1^, indicates the fastest OER kinetics. In general, it can judge the bifunctional catalytic activity of the material as a whole by the potential difference ΔE = E_j = 10_ (OER overpotential) – E_1/2_ (ORR half‐wave potential). The smaller the ΔE value, the better the bifunctional activity of the catalyst. As shown in Figure 4i, the ΔE value of 0.834 V for Fe–Co(DSA)@3DNC is much lower than that of Co(SA)@3DNC (0.928 V), Fe(SA)@3DNC (1.006 V), and Fe(SA)&Co(SA) (0.901 V), which indicates that Fe–Co(DSA)@3DNC material has excellent bifunctional catalytic activity.

### 2.4 Zinc–Air Battery Performance

**Figure 5:**
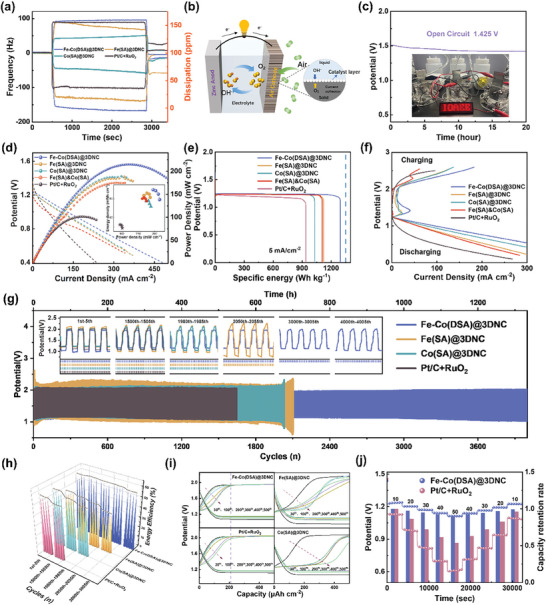
a) Q‐CMD test curve. b) Schematic diagram of Zinc–air battery. c) Open circuit voltage of Fe–Co (DSA)@3DNC (inset of self‐made Zinc–air battery lighting LED screen). d) Amplification curve and power density (inset of calculated energy density *vs*. power density). e) Energy density at 5 mA cm^‐2^. f) Charge–discharge polarization curve. g) Long cycle performance of Zinc–air batteries (inset of partial cycles). h) Charge–discharge energy efficiency. i) Charge–discharge polarization of different cycles. j) Rate performance test.

The adsorption and desorption of electrolytes play an important role in Zinc–air reactions, and high‐concentration electrolytes are easy to cause catalyst poisoning. Hence, the dissipative quartz crystal microbalances (QCM) test was employed to study the adsorption and desorption of ions by catalysts,^[^ ^29^ ^]^ as shown in **Figure** **5**a. The baseline is first determined in deionized water, and then 6.0 m KOH and 0.2 m (CH_3_COO)_2 _Zn electrolyte are introduced, at which point the frequency line decreases and the dissipation line rises significantly. It can be seen that Fe–Co(DSA)@3DNC has absorbed the maximum amount of electrolyte, which is attributed to its highest electrochemically active surface area. Besides, when the electrolyte is removed and the deionized water is re‐injected, the dissipation and frequency response of Fe–Co(DSA)@3DNC returns to the initial values, indicating that the ions could be fully desorption. Based on the excellent bifunctional OER/ORR catalytic activity of Fe–Co(DSA)@3DNC described above, it is used as a catalyst for air cathodes, with zinc plate as anode and 6.0 m KOH and 0.2 m (CH_3_COO)_2 _Zn as electrolytes to assemble a rechargeable ZAB (Figure 5b). Fe–Co(DSA)@3DNC based ZAB can maintain a stable open‐circuit voltage of 1.425 V after up to 20 h (Figure 5c), showing excellent resistance to self‐discharge. The power densities are calculated by the ex‐polarization curves, where Fe–Co(DSA)@3DNC catalyst has a high value of 215 mW cm^−2^, much higher than that of Fe(SA)@3DNC, Co(SA)@3DNC, Fe(SA)&Co(SA), and Pt/C&RuO_2_, as shown in Figure 5d. The instantaneous energy density of Fe–Co(DSA)@3DNC is also higher than other materials. Figure 5e shows the specific energy of the batteries. The specific energy value of Fe–Co(DSA)@3DNC is 1295 Wh kg^−1 ^_Zn_, which is much higher than Fe(SA)@3DNC, Co(SA)@3DNC, Fe(SA)&Co(SA), and Pt/C&RuO_2_ and close to the theoretical value of 1350 Wh kg^−1 ^_Zn_ (due to the influence of oxidation on the surface of zinc sheets, resulting in an overall high value).^[^ ^30^ ^]^ Compared with other contrast catalysts, Fe–Co(DSA)@3DNC has a relatively small difference in charge and discharge polarization voltage (see Figure 5f). Galvanostatic charge–discharge cycle performance of these catalysts at a current density of 5 mA cm^−2^ is shown in Figure 5g. The Fe–Co(DSA)@3DNC electrode exhibits excellent stability of >4000 cycles (1350 h), far exceeding Fe(SA)@3DNC, Co(SA)@3DNC, and Pt/C&RuO_2_ electrodes. Notably, Fe–Co(DSA)@3DNC electrode maintains a stable charge–discharge voltage from the start to the 4000^th^ cycle, and no polarization occurred, proving a highly reversible reaction without side reactions occurred. Post‐cycling XPS tests are provided to verify the excellent stability of the Fe–Co(DSA)@3DNC electrode, as shown in Figure S11 (Supporting Information). The tests are carried out directly on the electrodes rather than powder materials, so there is less catalyst on the surface of carbon paper, which is difficult to accurately characterize Fe and Co elements. However, it can be found from its full spectrum that no matter after 10, 50, and 3500 cycles, only a very small amount of K and Zn elements accumulate on the electrode. In addition, no impurities are found, which confirms its excellent cyclic stability.

Figure 5h shows the charge and discharge energy efficiencies of these electrodes. The Fe–Co(DSA)@3DNC electrode maintains from 58.50% at the beginning to 56.94% after 3000 cycles, while the efficiency of other electrodes decreases significantly as the number of cycles increases. In order to further analyze the reaction kinetics of electrodes, voltage–capacity curves are carried out as shown in Figure 5i. It can be seen that with the proceeding of the charge‐discharge reaction, the discharge curves of the four electrodes do not change much, while the charge curves change significantly. This phenomenon indicates that the OER reaction is much more difficult than the ORR reaction in ZABs, and the OER reaction may be one of the rate‐controlled steps. Among them, Fe–Co(DSA)@3DNC has the lowest charge–discharge polarization, proves its stability. Figure 5j shows the rate performance of the electrodes at current densities of 10 to 50 mA cm^−2^. The Fe–Co(DSA)@3DNC electrode possesses a discharge voltage of 1.1 V when the current density increases to 50 mA cm^−2^ with a high voltage retention rate of 89%, exhibits excellent rate performance, far exceeding that of Fe(SA)@3DNC, Co(SA)@3DNC, Fe(SA)&Co(SA), and commercial Pt/C&RuO_2_ electrodes (see Figure S12, Supporting Information). ZABs mainly discuss the reaction of oxygen, but the air has a high content of N_2_ and CO_2_, which may participate in the reaction or cause catalyst poisoning. Hence, the reaction of Fe–Co(DSA)@3DNC electrode in pure N_2_ or CO_2_ was tested. As can be seen in Figure S13 (Supporting Information), the reaction voltage far exceeds the range of the Zinc–O_2_ reaction, so N_2_ and CO_2_ will not have an impact on Fe–Co(DSA)@3DNC electrode (detailed discussion can be seen in Supporting Information). A comprehensive comparison of the electrochemical properties of Fe–Co(DSA)@3DNC with other similar works is shown in Figure S14 (Supporting Information), illustrating its superiority.

### 2.5 Seesaw Effect in Catalytic Mechanism

**Figure 6:**
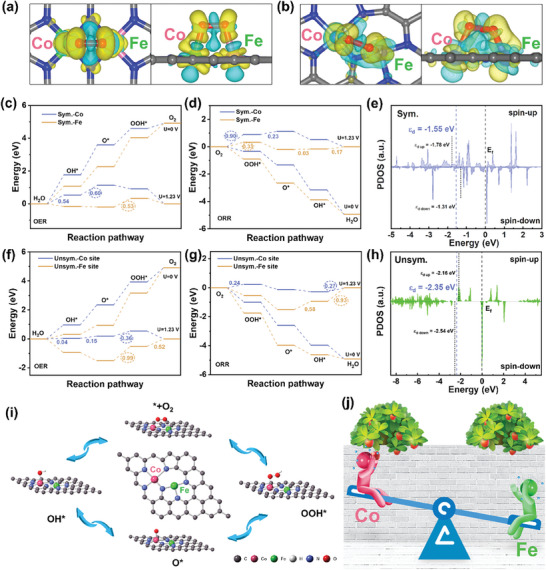
The charge density difference of a) the symmetric model and b) the unsymmetric model. c) OER and d) ORR free energy change plots of the Co site and Fe sites in the symmetric model. e) The projected density of states (DOS) of the symmetric model. f) OER and g) ORR free energy change plots of the Co sites and Fe sites in the unsymmetric model. h) The projected density of states (DOS) of the unsymmetric model. i) Schematic diagram of the OER/ORR reaction path of an unsymmetric Fe–Co(DSA)@3DNC. (j) The seesaw effect between Fe and Co sites.

In order to further study the ORR/OER active site and reaction mechanism of this special unsymmetric Fe–Co bimetallic pair model in ZAB. DFT calculation was carried out. As a comparison, a symmetric Fe–Co coordination structure model is also established. The differential charge density distribution plot of **Figure** **6**a,b shows the charge transfer between Fe–Co atoms. The yellow and cyan colors represent the electron‐rich and depletion regions, respectively. In the symmetric model (Figure 6a), the charge distribution between Fe and Co is relatively uniform. While, in the unsymmetric model (Figure 6b), the charge distribution between Fe and Co has a large polarization, and the delocalized electrons transfer and aggregate from Fe atom to Co atom, which is consistent with the results obtained by XPS and XANES. Thereafter, the free energy calculation of the ORR/OER process was carried out on the two models with Fe and Co as the active sites for an in‐depth study.^[^ ^31^ ^]^ Figure 6c shows the free energy diagram of OER process at Fe and Co positions in the symmetric model respectively. Since OER is a semi‐reaction of an electrolytic cell, that is, a charging reaction, when the external potential (U) is 0, the reaction is endothermic. It is known that the equilibrium ionization potentials of the ideal OER and ORR reactions are both 1.23 V, and the energy efficiency of the ideal catalyst is 100%. However, in reality, according to the second law of thermodynamics, the ionization potential of the catalyst cannot reach 1.23 V. So, when U = 1.23 V, taking the Co site in the symmetric model as an example, the step from OH^*^ to O^*^ needs to absorb 0.60 eV, and the remaining steps do not need to endothermic, or the value of absorption is <0.60 eV. Since the OER reaction undergoes an electron transfer at each step, the overpotential of the entire OER reaction at the Co site is μ = 0.60 V, which means that at least 1.23 V + 0.60 V = 1.83 V voltage needs to be applied to activate the OER reaction at the Co site. Similarly, it can be concluded that the overpotential of Fe site in the symmetric model is μ = 0.53 V, which is less than 0.60 V of Co site. Therefore, the Fe site in the symmetry model is the main active site for OER, with an overpotential of μ = 0.53 V. The schematic diagram of the reaction intermediate states of each active site is shown in Figures S15–S18 (Supporting Information). Figure 6d shows the free energy diagram of ORR process at Fe and Co positions in the symmetric model respectively. Same as above, the overpotentials of the entire ORR reaction at Co site and Fe site are 0.90 and 0.33 V (from O_2_ to OOH^*^), respectively. Therefore, the Fe site in the symmetry model is the main active site for ORR, and the overpotential is μ = 0.33 V. When it comes to the unsymmetric model this work obtained, the overpotential of the OER process at Co site is μ = 0.36 V, much lower than the lowest overpotential of 0.53 V (Fe site) in the symmetric model. Meanwhile, the overpotential of the ORR process at Co site is μ = 0.27 V, much lower than the lowest overpotential of 0.33 V (Fe site) in the symmetric model. Therefore, in the symmetric model, the Fe site plays an important role in whole OER/ORR, while in the unsymmetric model, the Co site plays an important role and far exceeds the Fe site in the symmetric model. In this regard, Figures 6e,h show the projected state densities of the d‐orbital of the symmetric model and the unsymmetric model, respectively. The d‐band center (−2.35 eV) of the unsymmetric model moves negatively compared to that of the symmetric model (−1.55 eV). It means that there are more electrons occupying the anti‐bonding orbitals in the unsymmetric model, resulting in a weaker bond strength with oxygen intermediates, which is more conducive to the dissociation of the intermediates. The number of electrons in different spin states of each atom was also calculated (see Figure S19, Supporting Information). In the symmetric model, Fe and Co exhibit a similar electron distribution, with more spin‐up electrons (4.36 for Co and 4.37 for Fe) than spin‐down electrons (3.59 for Co and 3.14 for Fe). However, in the unsymmetric model, electron orbitals are rearranged and the electron distribution of the Co site is abnormal. The number of spin‐down electrons (4.73) at Co site increases significantly, far more than the number of spin‐up electrons (2.58), showing a large spin polarization. This may be one of the reasons for the improvement of Co catalytic performance. Electron paramagnetic resonance (EPR) was used to investigate the spin polarization among the three catalysts, as shown in Figure S20 (Supporting Information). Generally speaking, low‐spin Fe–N–C or Co–N–C species have very few unpaired electrons, so the signal‐to‐noise ratio of the catalysts is very poor. After proper smoothing, it can be seen weak symmetric peaks at g = 1.98, corresponding to delocalized electrons. The total number of delocalized electrons in Fe–Co(DSA)@3DNC is the largest, which supports the theoretical calculation results.

Combined with the results of XAFS, the analysis shows that there is a special weak interaction between Fe and Co, which can be considered as spin polarization bonding.^[^ ^32^ ^]^ Overall, the OER and ORR reaction paths that occur in Fe–Co(DSA)@3DNC are shown in Figure 6i. Fe–Co bimetallic atom pair in the unsymmetric model form a seesaw effect, as shown in Figure 6j. Fe atoms provide delocalized electrons to Co atoms, forming electron‐rich regions at Co atoms, improving the catalytic performance of Co atoms, and finally reducing the overpotential of the whole reaction. In addition, the electron transfer between Fe and Co rearranges the electron orbitals, which lowers the d‐band center and promotes the desorption of intermediates. Therefore, the electrochemical reaction kinetics of Fe–Co(DSA)@3DNC are jointly enhanced from the perspectives of “adsorption‐catalysis‐desorption”.

### 2.6 Flexible Zinc–Air Battery Performance

**Figure 7:**
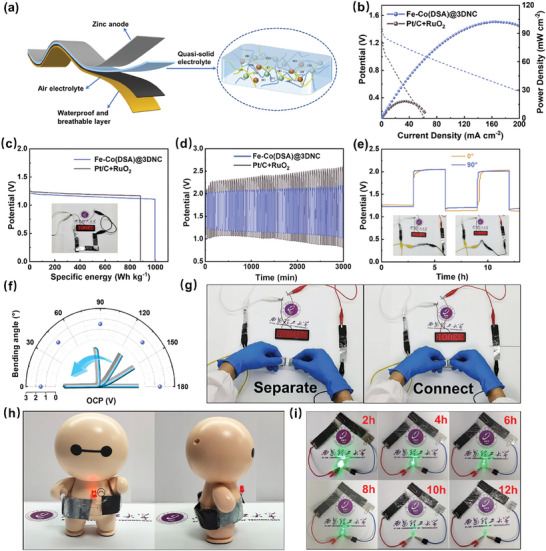
a) Schematic diagram of the flexible Zinc–air battery. b) Discharge polarization curve and power density. c) Specific energy density (inset of flexible Zinc–air battery lighting LED screen). d) Long cycle performance. e) Charge–discharge curve at original and bent state (inset is a lighting experiment in two states). f) Open‐circuit voltage of flexible Zinc–air battery at different bending angles. g) Shear and self‐healing performance of the flexible battery. h) A doll wearing a flexible battery can successfully power the LED lamp. i) The green LED is powered by two flexible batteries for 12 h.

Flexible wearable energy storage has been a research trend in recent years.^[^ ^33^ ^]^ Herein, Fe–Co(DSA) @3DNC‐based flexible ZAB was also assembled with a quasi‐solid electrolyte, as shown in **Figures** **7**a and S21 (Supporting Information). As can be seen in Figure 7b,c, the Fe–Co(DSA) @3DNC‐based flexible ZAB still delivers a peak power density of 102.5 mW cm^−2^ and a specific energy of 997 Wh kg^−1 ^_Zn_, which is much higher than the 32 mW cm^−2^ and 880 Wh kg^−1 ^_Zn_ of Pt/C&RuO_2_ electrode. The flexible ZAB also has a cycling performance of more than 3000 min (see Figure 7d). Besides, bending and rotating has no effect on the charge–discharge curve of the flexible battery (see Figure 7e). Regardless of any bending angle applied, the discharge voltage of the flexible battery is always maintained at 1.39 V, demonstrating its potential in the field of wearable energy storage (see Figure 7f). Under emergency conditions, the battery may be physically damaged. Figure 7g shows the self‐healing function of the flexible battery. Safety is the most important indicator of wearable devices. In this flexible battery, all components are nonflammable and a short period of high‐temperature combustion will not affect the battery (see Figure S22, Supporting Information). Finally, the flexible battery is wrapped around a doll for use, and it can light an LED lamp for >10 h.

## 3 Conclusion

In conclusion, an unsymmetric Fe–Co bimetallic single‐atom catalyst is developed via the assistance of an ionic liquid. Based on the adsorption‐catalysis‐dissociation mechanism, the reaction kinetics were optimized and excellent electrochemical performance was achieved, including a peak power density of 215 mW cm^−2^ and stable cycling for >1300 h and >4000 cycles. This can be attributed to the following key innovations: (i) the unsymmetric configuration of Fe and Co sites forms a strong charge polarization between them due to electron transfer, which enhances the delocalization of d‐band electrons and significantly accelerates the electron conduction, (ii) the electrons spin up and down at the Co site show an unsymmetric distribution, and the spin polarization produces a stray field, which optimizes the d‐band center of the central atom. It weakens the adsorption of oxygen intermediates in the OER/ORR reaction, maintains the mildness of the interaction force, balances the adsorption‐dissociation relationship, and ultimately reduces the reaction energy barrier, (iii) the assistance of ionic liquids enhances the anchoring of atoms in the unsymmetric configuration of Fe–Co(DSA)@3DNC, ensuring the stability of the micro‐nano structure after repeated adsorption‐catalysis‐dissociation of oxygen intermediates. The results of this work can inspire more attention to the tip effect in catalytic sites, rather than using each site in a balanced manner.

## Conflict of Interest

The authors declare no conflict of interest.

## Supporting information

Supporting Information

## Data Availability

The data that support the findings of this study are available from the corresponding author upon reasonable request.
