# Supplementary material for: Electron Spin Broken‐Symmetry of Fe–Co Diatomic Pairs to Promote Kinetics of Bifunctional Oxygen Electrocatalysis for Zinc–Air Batteries
Source: Adv Sci (Weinh). 2024 Jun 14;11(35):2401187. doi: 10.1002/advs.202401187 (PMC11425208; doi:10.1002/advs.202401187)
Supplement: Supplementary file 1 — Supporting Information [file ADVS-11-2401187-s001.pdf]

Supporting Information

Electron Spin Broken-Symmetry of Fe-Co Diatomic Pairs to Promote Kinetics of Bifunctional Oxygen Electrocatalysis for Zinc-Air Batteries

Xiaokang Li [a, b], *Jian Qin** [a, b, c], *Qingxin Lin* [a, b], *Xiaoyu Yi* [a, b], *Cheng Yan* [a, b], *Jianhua Zhang* [a, b], *Jinjuan Dong* [a, b], *Kang Yu* [a, b], *Shenglong Zhang* [a, b], *Chong Xie* [a, b], *Huijuan Yang* [a, b], *Wei Xiao* [a, b], *Wenbin Li* [a, b], *Jingjing Wang* [a, b], *Xifei Li** [a, b]

a. Institute of Advanced Electrochemical Energy & School of Materials Science and Engineering, Xi'an University of Technology, Xi'an, Shaanxi, 710048, China

b. Shaanxi international Joint Research Center of Surface Technology for Energy Storage Materials, Xi'an, Shaanxi, 710048, China

c. Department of Materials Science and Engineering, Macau University of Science and Technology, Macau, 999078, China

E-mail: qinjian@xaut.edu.cn; xfli@xaut.edu.cn

Experimental Section

Preparation of Fe-Co(DSA)@3DNC:

The reagents were purchased from Aladdin Chemical Reagent Company and used without further purification. Firstly, 0.054 g $FeCl_3$•$6H_2O$, 0.058 g $Co(NO_3)_2$•$6H_2O$, 4.28 g ammonium citrate, 0.68 g melamine and 25g NaCl were dissolved in 400 ml distilled water and stirred for 5 hours. Next, the resulting solution is placed at a low temperature to ensure that it freezes completely into ice, and then subjected to freeze-drying. Afterward, the precursor was placed in a tubular furnace and held at 1000 degrees Celsius for 2 hours in argon atmosphere. Finally, the NaCl was repeatedly cleaned with deionized water to obtain the catalyst named Fe-Co(DSA)@3DNC. For comparison, Fe and Co monmetallic monatomic catalysts were prepared by the same method,

denoted as Fe(SA)@3DNC and Co(SA)@3DNC.

Materials Characterization:

XRD (*/SmartLab), SEM (ZEISS, MERLIN Compact) TEM (JEOL JEM-2100Plus) and HADDF scanning transmission electron microscopy (JEM-ARM200F) were applied to analyze the crystalline phases, morphology, and nanostructure of obtained samples. The contents of Co and Fe were determined by inductively coupled plasma (ICP). Raman (HR Evolution) and XPS (Thermo Fisher) were taken to investigate the surface states. The X-ray absorption spectra were collected on the beamline BL01C1 in NSRRC. Electrochemical quartz crystal microbalance (EQCM) measurements were conducted by a GAMRY (Warminster, PA) EQCM system.

Electrochemical Measurements:

Electrochemical measurements were carried out at room temperature in the standard three-electrode system. The electrochemical workstation was CHI 760E and Autolab PGSTAT302N. The working electrode was glassy carbon rotating disk electrode. In 0.1 M KOH solution, the reference electrode and counter electrode was saturated calomel electrode and graphite sheet, respectively. The reference electrode is calibrated before testing. ORR catalyst ink is synthesized as follows: 3 mg catalyst is dispersed in a solution consisting of 25 μL Nafion, 225 μL ultra-pure water, and 250 μL ethanol. Then, the prepared catalyst ink was evenly coated on the working electrode, and was fully dried for further testing in 0.1M KOH. For comparison, we tested commercial Pt/C (20 wt%) catalyst with a fixed load of 0.6 mg cm^{-2}. OER catalyst ink is synthesized as follows: 4 mg catalyst is dispersed in a solution consisting of 50 μL Nafion, 500 μL ultrapure water, and 500 μL ethanol. Then, the prepared catalyst ink was evenly coated on the working electrode. After being fully dried, it was further tested in 1 M KOH. For comparison, we tested the performance of commercial RuO_2 with a fixed load of 0.194 mg cm^{-2}. The LSV curves are IR corrected by CHI760E electrochemical workstation. The compensation level is 80%. The potentials (vs SCE) measured in this work are converted to reversible hydrogen electrode potential (RHE) and corrected according to the Nernst equation. For zinc-air batteries, the catalyst ink was obtained by dispersing 10 mg of catalyst in a solution composed of 100 μL Nafion,

700 μL ultra-pure water and 700 μL ethanol. The catalyst ink was evenly coated on a carbon paper with a loading of 1 mg cm^{-2}. The polished zinc plate was used as the anode, and the mixed solution of 6M KOH and 0.2M $(CH3COO)_2Zn$ was used as the electrolyte to assemble the Zinc-air batteries. The electrochemical tests of Zinc-air batteries were performed on Autolab (PGSTAT302N, Metrohm China Ltd.) electrochemical workstation and NEWARE battery testers. Under the above conditions, a mixture of Pt/C (20wt%) and RuO_2 (in a ratio of 1:1) was used as a contrast catalyst. For flexible zinc-air batteries, the PANa-based hydrogel electrolyte was soaked in 6M KOH + 0.2M $(CH3COO)_2Zn$ electrolyte.

Density functional theory calculations.

Density functional theory (DFT) calculations were performed using the quantum espresso (QE) [1, 2] based on the pseudopotential plane wave (PPW) method. The perdew-Bueke-Ernzerhof (PBE) functional [3] was used to describe exchange-correlation effects of electrons. We have chosen the projected augmented wave (PAW) potentials[4, 5] to describe the ionic cores and take valence electrons into account using a plane wave basis set with a kinetic energy cutoff of 500 eV. All structures were first optimized to reach their most stable configuration. During the geometry optimizations, all atom positions were allowed to relax.

The Gibbs free energies were calculated as following: $\Delta G=\Delta E_{DFT}+\Delta ZPE-T_{\Delta S}$ where E_{DFT}, ZPE and S were DFT calculated electronic energies, zero point energies and entropies of the molecules obtained from harmonic oscillation approximation and standard thermodynamic tables, respectively.

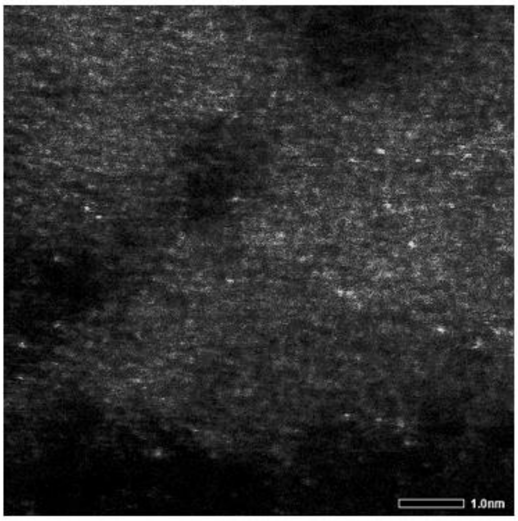

Figure S1 The ACTEM image before filtering.

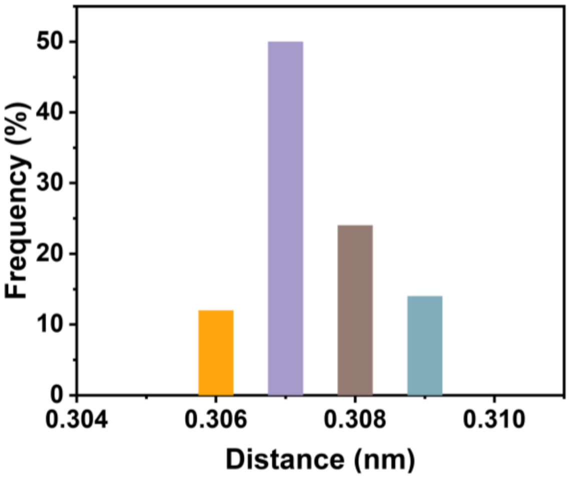

Figure S2 Statistical results of the distance between diatomic pairs.

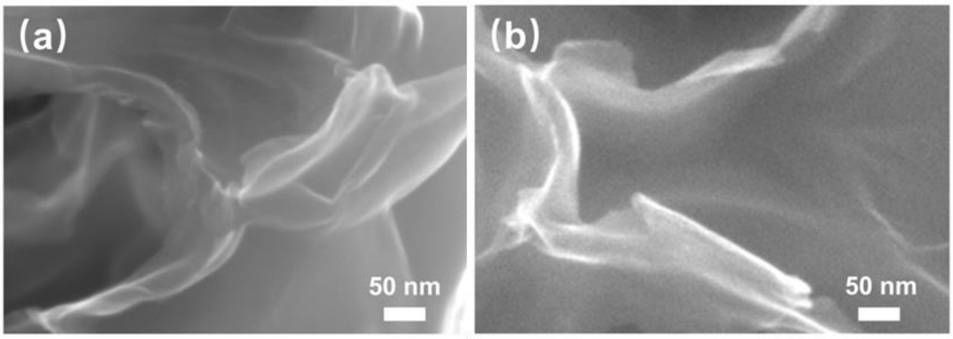

Figure S3 SEM image of (a) Fe(SA)@3DNC and (b) Co(SA)@3DNC.

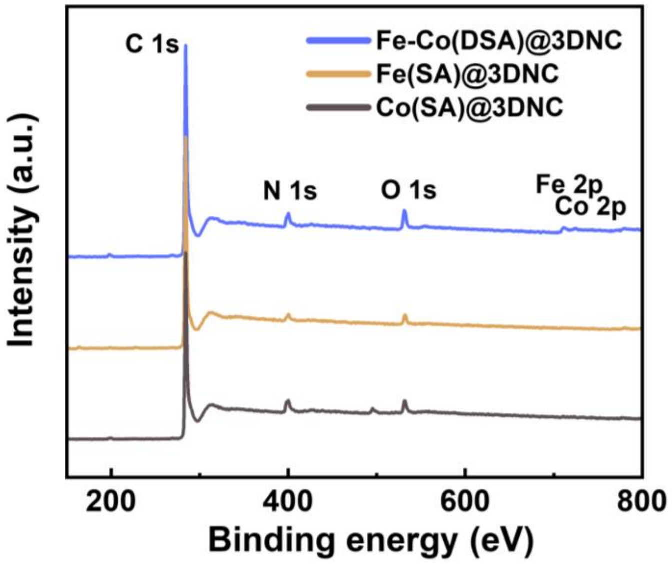

Figure S4 XPS spectra of Fe-Co(DSA)@3DNC, Fe(SA)@3DNC and Co(SA)@3DNC.

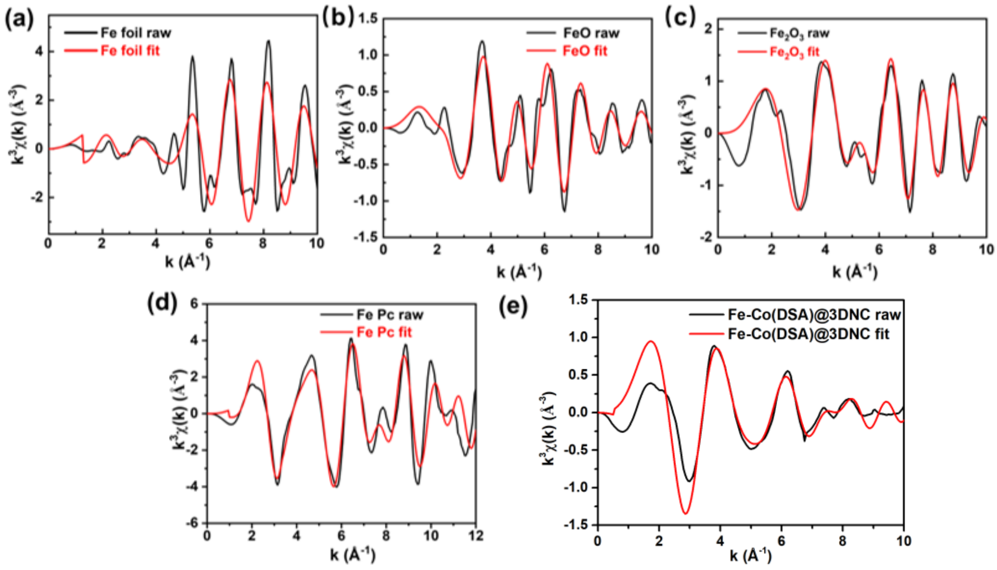

Figure S5 Fe K-edge EXAFS analysis of (a) Fe foil, (b) FeO, (c) Fe_2O_3, (d) FePc and (e) Fe-Co(DSA)@3DNC in κ spaces.

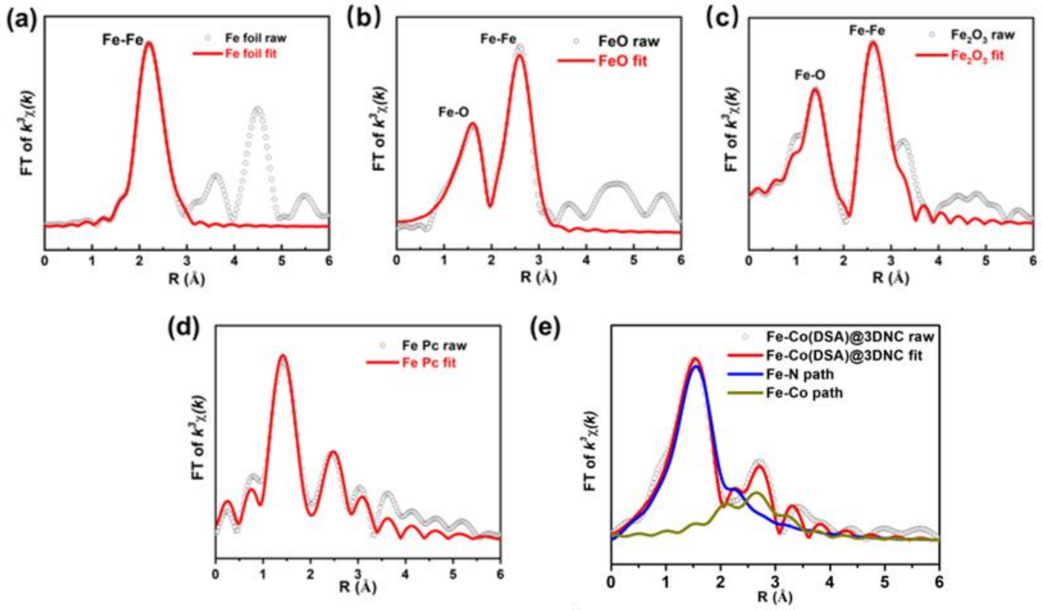

Figure S6 Fe K-edge EXAFS analysis of (a) Fe foil, (b) FeO, (c) Fe_2O_3, (d) FePc and (e) Fe-Co(DSA)@3DNC in R spaces.

It can be seen in Fe K-edge EXAFS analysis of Fe-Co(DSA)@3DNC in κ spaces that the result is very similar to FePc or Fe_2O_3. While, R spaces result shows that there is no Fe-Fe peak in Fe-Co(DSA)@3DNC and its main peak is the same as that of FePc, , proving the coordination of Fe-N.

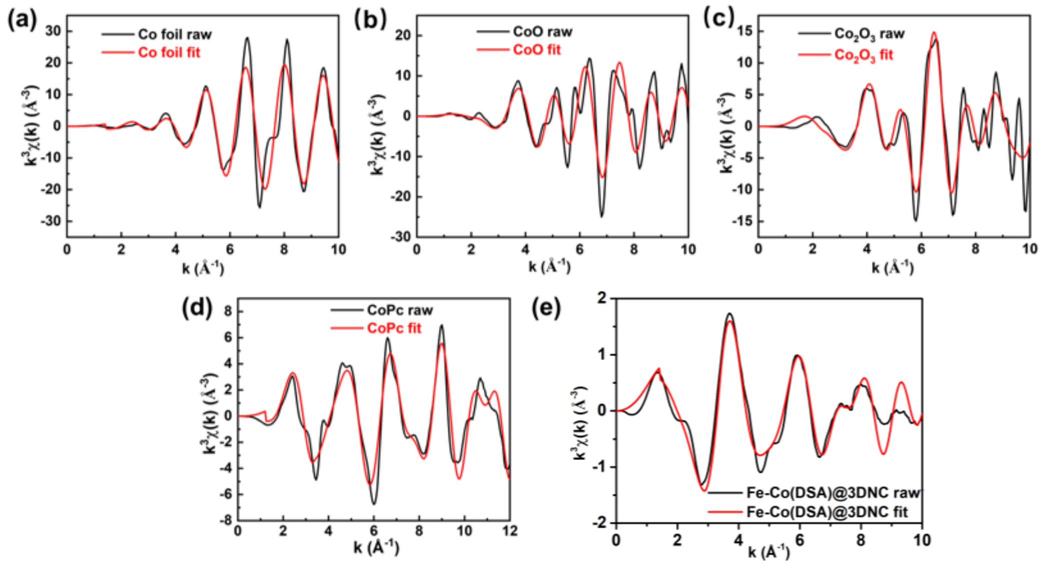

Figure S7 Co K-edge EXAFS analysis of (a) Co foil, (b) CoO, (c) Co_2O_3, (d) CoPc and (e) Fe-Co(DSA)@3DNC in κ spaces.

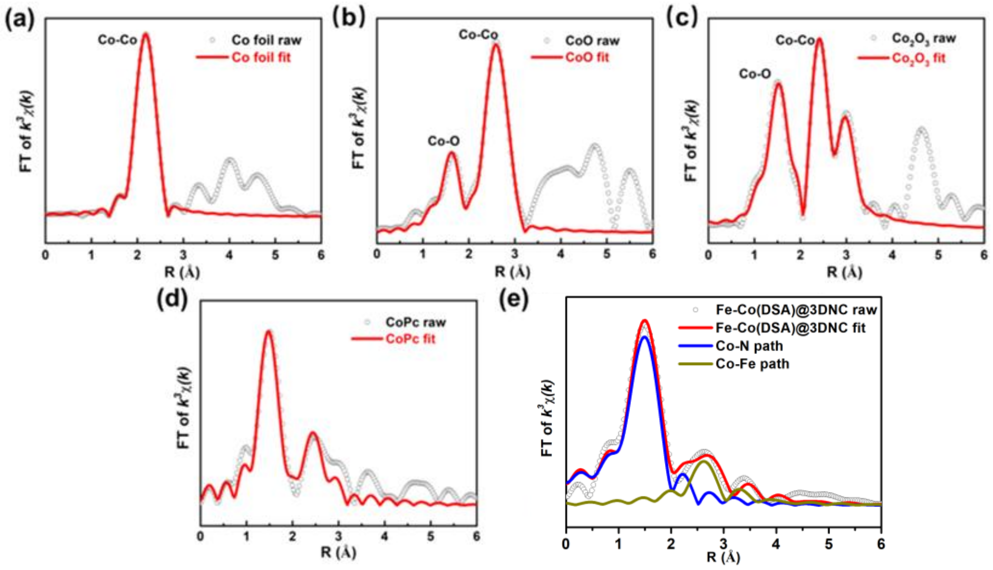

Figure S8 Co K-edge EXAFS analysis of (a) Co foil, (b) CoO, (c) Co_2O_3, (d) CoPc and (e) Fe-Co(DSA)@3DNC in R spaces.

It can be seen in Co K-edge EXAFS analysis of Fe-Co(DSA)@3DNC in κ spaces that the result is very similar to CoPc or CoO. While, R spaces result shows that there is no Co-Co peak in Fe-Co(DSA)@3DNC and its main peak is the same as that of CoPc, proving the coordination of Co-N.

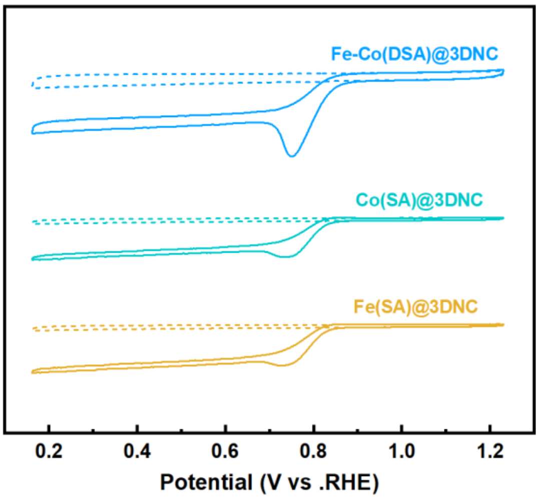

Figure S9 CV curves of Fe-Co(DSA)@3DNC, Fe(SA)@3DNC and Co(DSA)@3DNC in Ar-saturated electrolyte and O_2-saturated electrolyte.

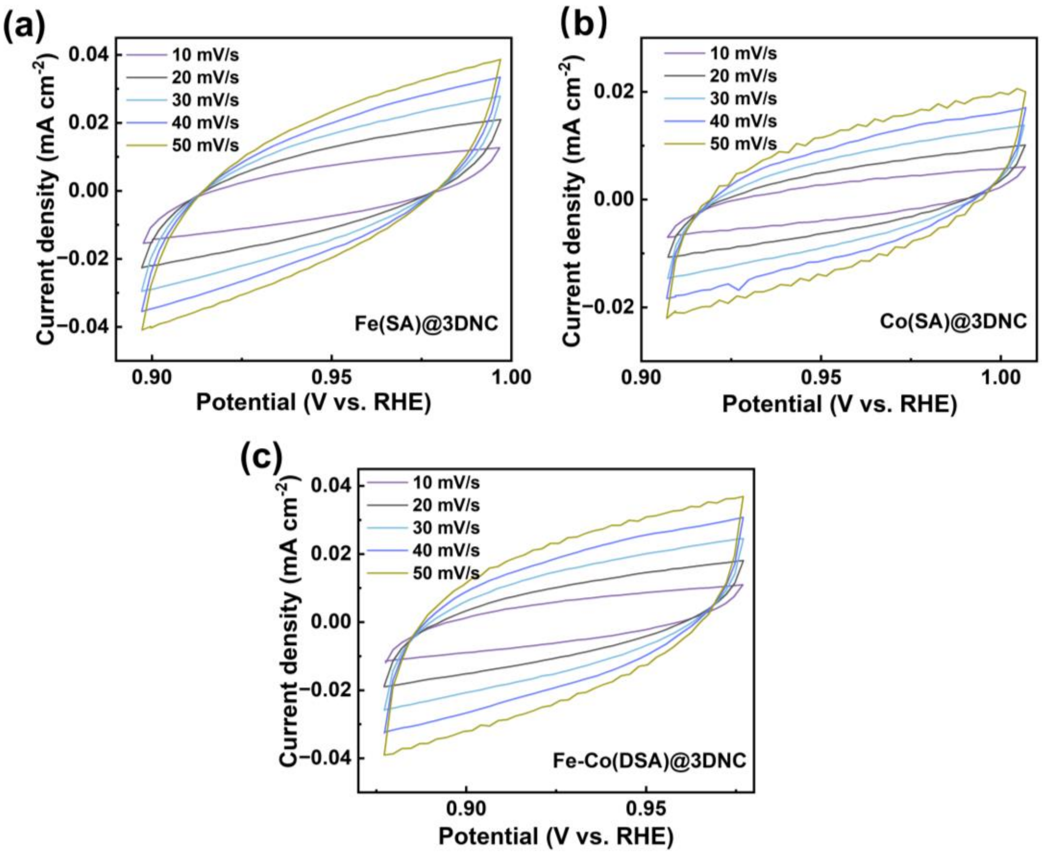

Figure S10 Multi-scan CV curves of (a) Fe(SA)@3DNC, (b) Co(SA)@3DNC and (c) Fe-Co(DSA)@3DNC.

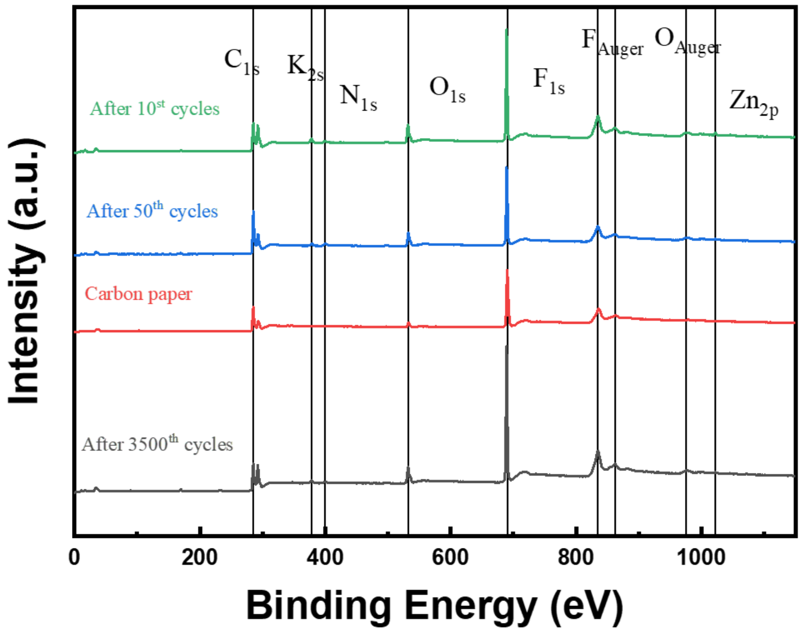

Figure S11 The post-cycling XPS spectra of Fe-Co(DSA)@3DNC.

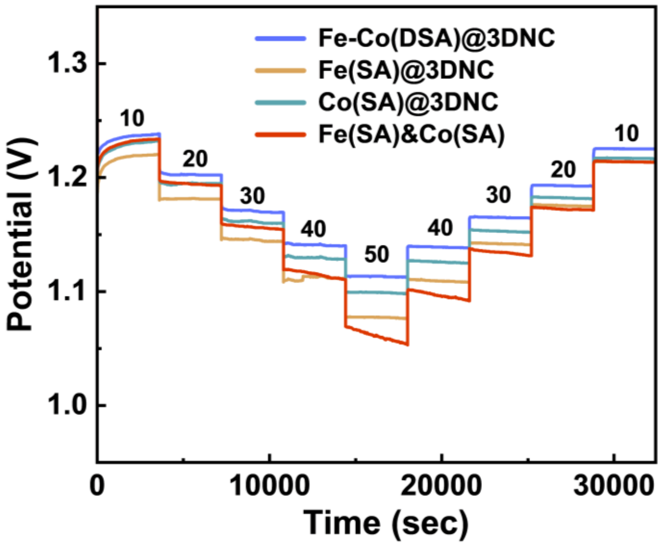

Figure S12 Rate performance of Fe-Co(DSA)@3DNC, Fe(SA)@3DNC, Co(SA)@3DNC and Fe(SA)&Co(SA).

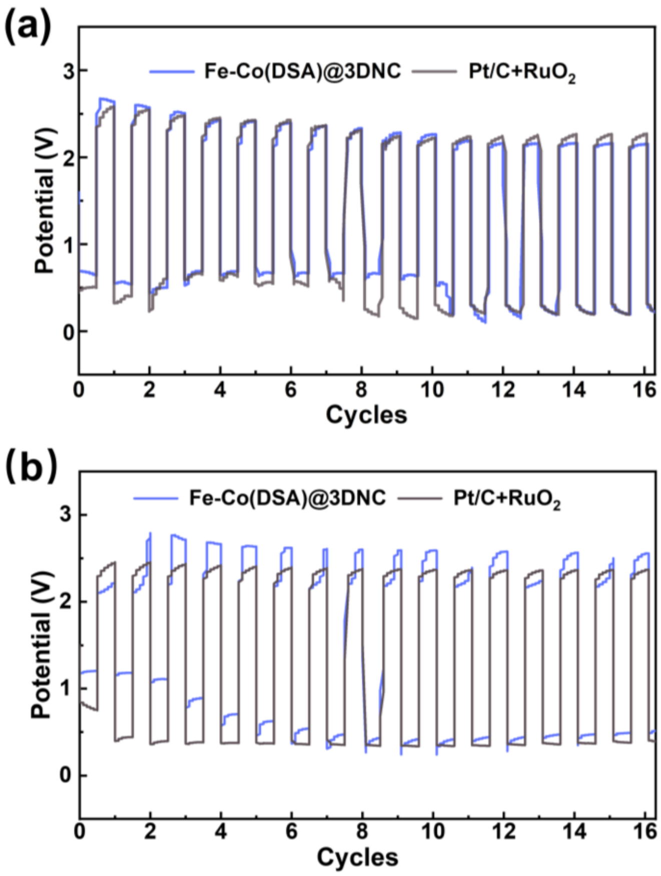

Figure S13 Charge-discharge curve tested in (a) CO_2 and (b) N_2 atmosphere.

It can be seen that Fe-Co(DSA)@3DNC has capacity when tested in N_2 or CO_2 atmosphere, and can undergo charge-discharge cycles, proving its ability to catalyze the redox reaction of N_2 and CO_2. However, its charge-discharge platform is extremely unstable, and the discharge platform (below 0.5 V *vs.* Zn) and charge platform (above 2.5 V *vs.* Zn) far exceed the reaction range of Zinc-O_2. Therefore, in this experiment, N_2 and CO_2 will not participate in the reaction and will not cause side reactions such as catalyst poisoning.

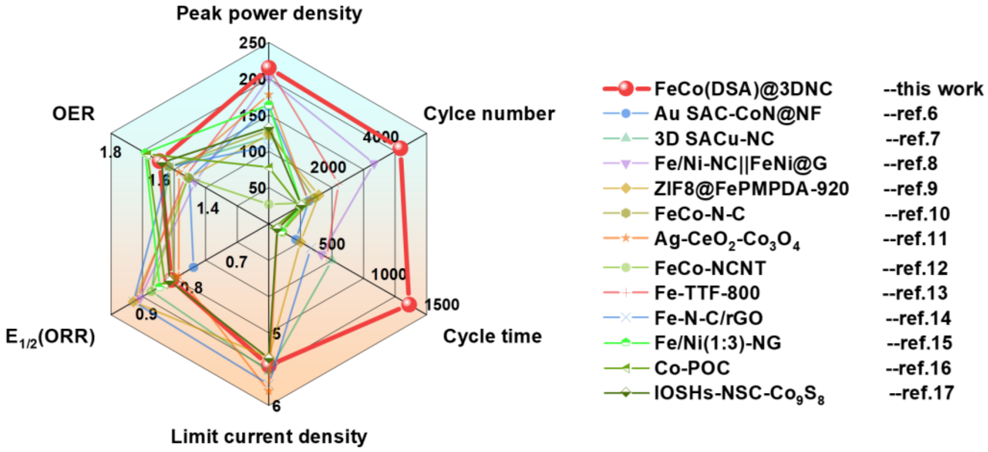

Figure S14 A comprehensive comparison of the electrochemical properties of Fe-Co(DSA)@3DNC with other similar works [6-17]

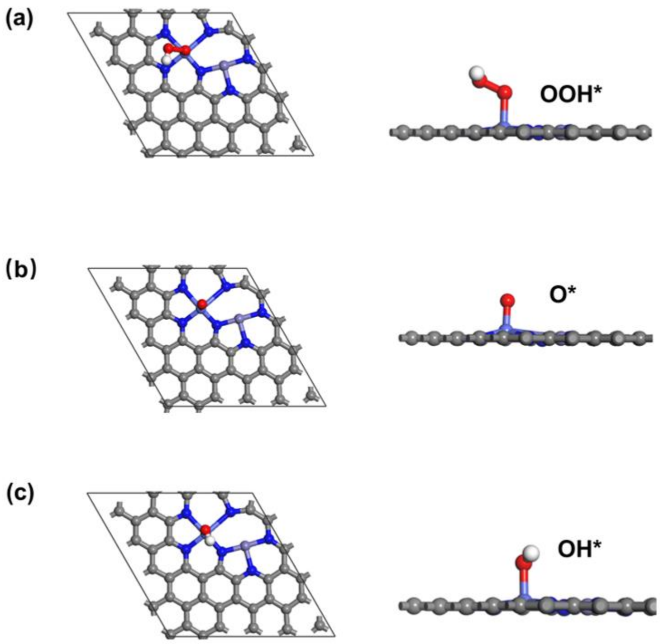

Figure S15 (a) OOH*, (b) O* and (c) OH* intermediate structure on Fe-Co(DSA)@3DNC (unsymmetric models) at Co site.

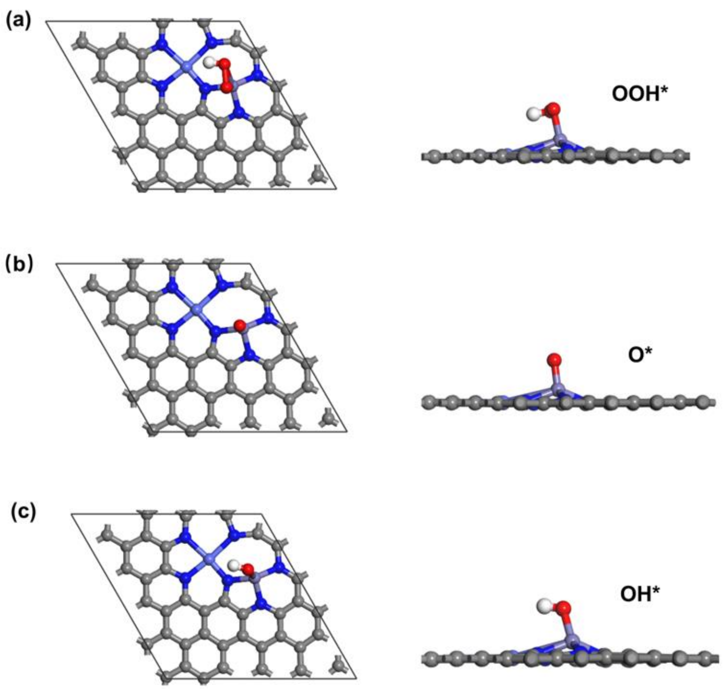

Figure S16 (a) OOH*, (b) O* and (c) OH* intermediate structure on Fe-Co(DSA)@3DNC (unsymmetric models) at Fe site.

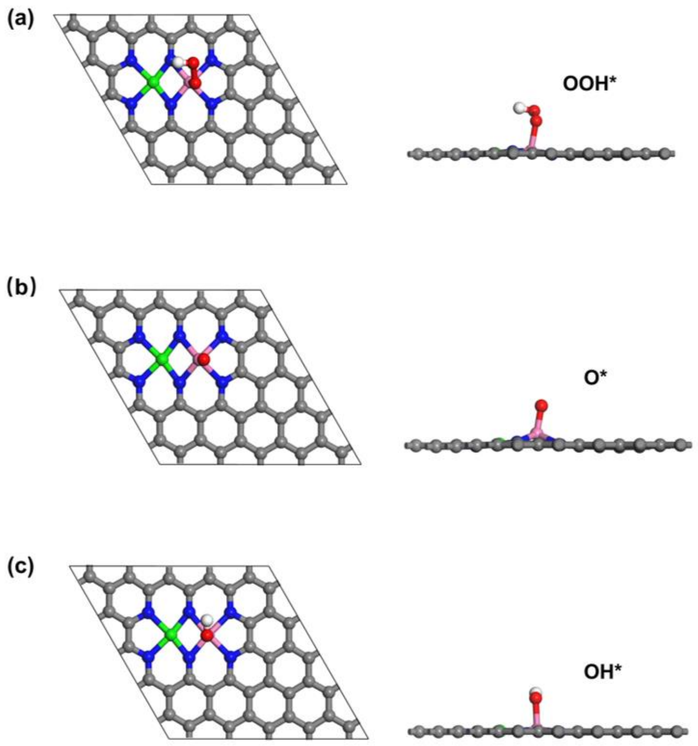

Figure S17 (a) OOH*, (b) O* and (c) OH* intermediate structure on symmetric models at Co site.

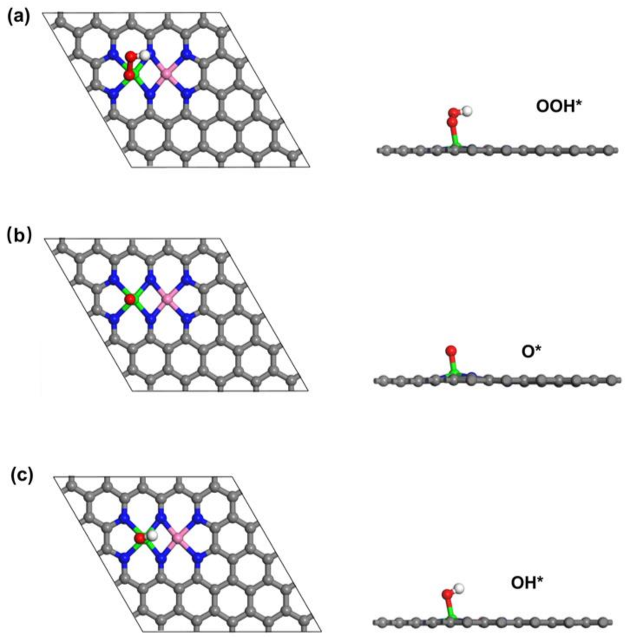

Figure S18 (a) OOH*, (b) O* and (c) OH* intermediate structure on symmetric models at Fe site.

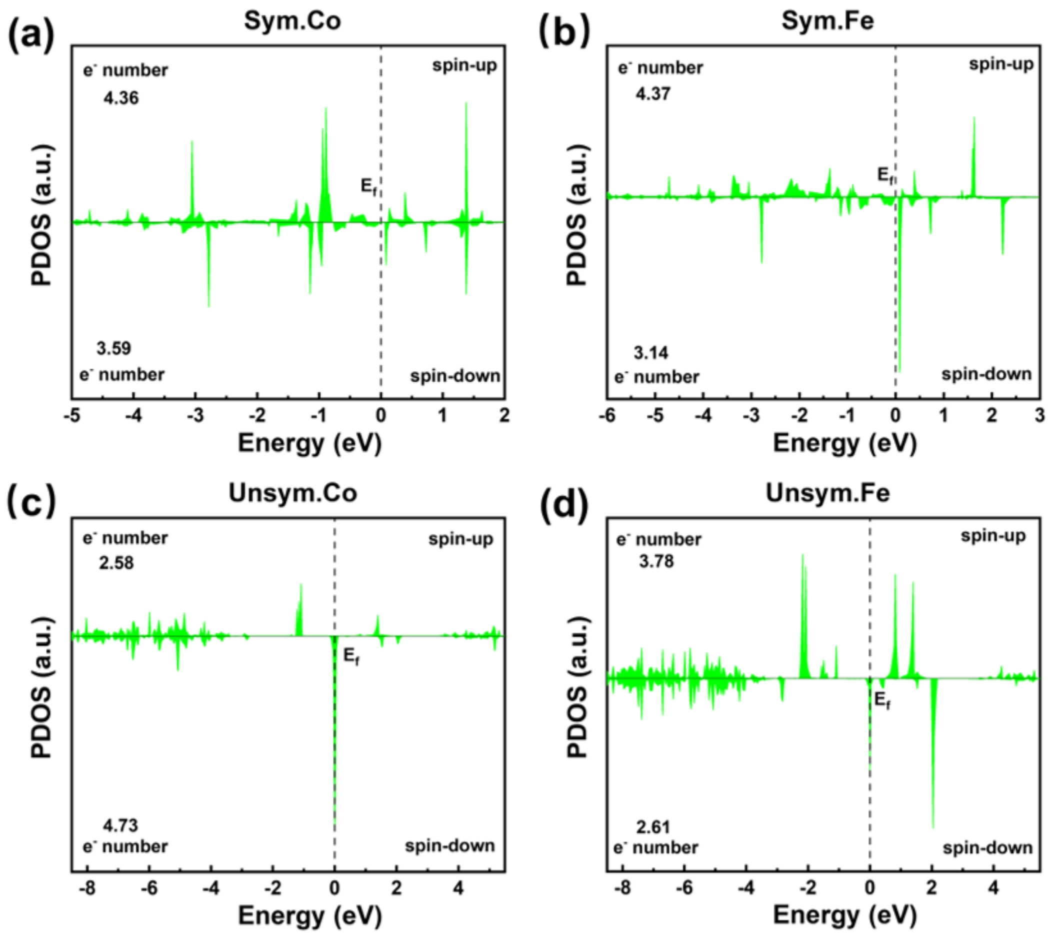

Figure S19 PDOS plots of (a) Co and (b) Fe in symmetric model. PDOS plots of (c) Co and (d) Fe in unsymmetric model.

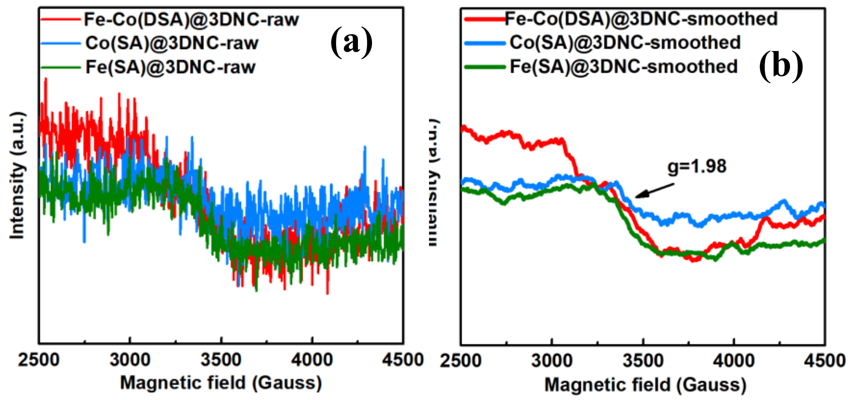

Figure S20 Room-temperature EPR spectra of Fe-Co(DSA)@3DNC, Co(SA)@3DNC and Fe(SA)@3DNC.

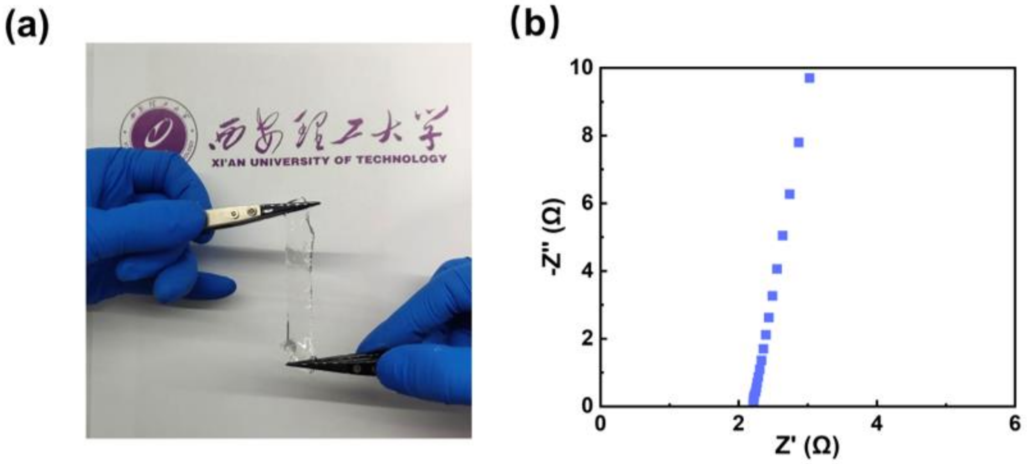

Figure S21 (a) Picture of PANa-based gel electrolyte. (b) Nyquist plot of gel electrolyte.

The gel electrolyte has certain toughness and its ionic conductivity is 68.9 mS cm^{-1}.

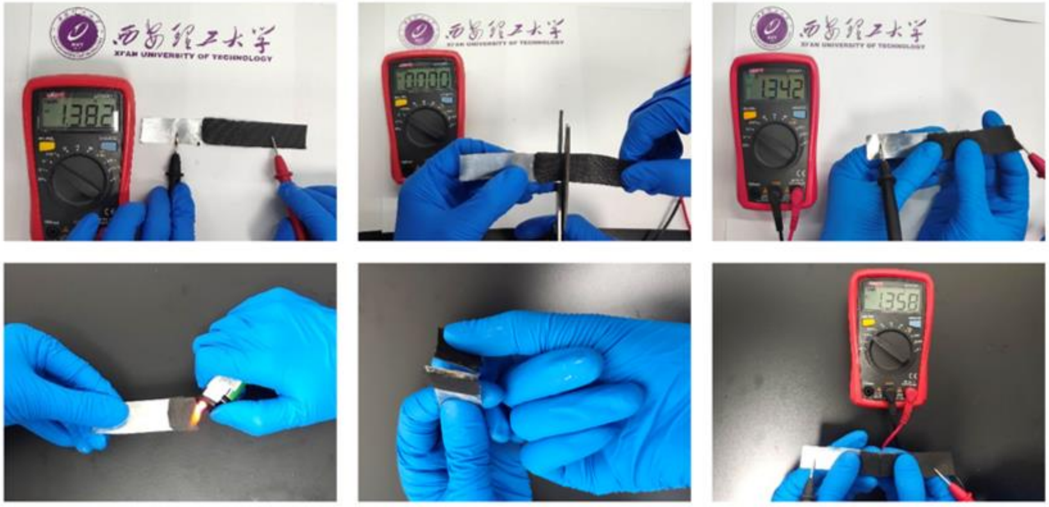

Figure S22 The flexible Zinc-air battery is sheared and burned by fire, and still has a stable open-circuit voltage.

In order to verify the potential application of the flexible battery under extreme conditions, shear and burning experiments were carried out. It can be seen that after the battery is cut and reconnected, its voltage only drops from 1.382 to 1.342 V. Moreover, burning the cross section of the cut battery with fire has no effect on its voltage.

Table S1 Comparison of the element contents in Fe-Co(DSA)@3DNC, Fe(SA)@3DNC and Co(SA)@3DNC catalysts obtained from XPS.

Sample	C (at%)	N (at%)	Fe (at%)	Co (at%)
**Fe-Co(DSA)@3DNC**	95.55	3.9	0.27	0.29
**Fe(SA)@3DNC**	93.5	5.91	0.59	-
**Co(SA)@3DNC**	93.83	5.52	-	0.66

Table S2 Comparison of the element contents in Fe-Co(DSA)@3DNC, Fe(SA)@3DNC and Co(SA)@3DNC catalysts obtained from ICP-MS.

Sample	Fe (wt%)	Co (wt%)
**Fe-Co(DSA)@3DNC**	1.93	1.20
**Fe(SA)@3DNC**	2.99	-
**Co(SA)@3DNC**	-	2.925

Table S3. EXAFS fitting parameters at the Co K-edge various samples (S_0^2=0.95).

samples	path	C. N.[a]	R (Å) [b]	σ^2 (× 10^{-3} Å^2) [c]	ΔE (eV) [d]	R factor[e]
Co foil	Co-Co	12*	2.51*	6.3±0.7	6.1±1.5	0.01
CoO	Co-O	6*	2.13*	5.7±3.5	-3.3±1.2	0.01
	Co-O-Co	12*	3.01*	9.4±1.8		
Co_2O_3	Co-O	3*	1.96*	2.1±0.5	-1.3±7.2	0.01
	Co-O	3*	2.27*	8.2±1.1		
	Co-O-Co	5*	3.06*	2.3±1.4		
	Co-O-Co	2*	3.26*	11.8±4.4		
Fe-Co(DSA)@3DNC	Co-N	3.8	2.09±0.02	6.8±4.8	-2.1±2.8	0.02
	Co-Fe	0.5	3.19±0.03	7.2±5.4		

^{a}C. N.: coordination numbers; ^bR: bond distance; $^c\sigma^2$: Debye-Waller factors; $^d\Delta E_0$: the inner potential correction. ^eR factor: goodness of fit. *The experimental EXAFS fit by fixing C. N. as the known crystallographic value.

Table S4. EXAFS fitting parameters at the Fe K-edge various samples (S_0^2=0.89).

samples	path	C. N.[a]	R (Å) [b]	σ^2 (× 10^{-3} Å^2) [c]	ΔE (eV) [d]	R factor[e]
Fe foil	Fe-Fe	8*	2.48*	6.4±1.3	6.6±1.3	0.01
	Fe-Fe	6*	2.87*	7.5±3.2		
FeO	Fe-O	6*	2.15*	8.4±5.9	-5.4±1.6	0.01
	Fe-Fe	12*	3.05*	9.7±6.2		
Fe_2O_3	Fe-O	3*	1.98*	6.1±1.7	-4.1±2.8	0.03
	Fe-O	3*	2.13*	8.3±3.5		
	Fe-Fe	3*	3.00*	8.1±2.2		
	Fe-Fe	3*	3.42*	11.6±3.8		
Fe-Co(DSA)@3DNC	Fe-N	3.1	2.04±0.02	7.8±3.1	1.0±4.4	0.02
	Fe-Co	1.2	3.14±0.02	6.6±5.2		

^{a}C. N.: coordination numbers; ^bR: bond distance; $^c\sigma^2$: Debye-Waller factors; $^d\Delta E_0$: the inner potential correction. ^eR factor: goodness of fit. *The experimental EXAFS fit by fixing C. N. as the known crystallographic value.

Reference:

[1] P. Giannozzi, S. Baroni, N. Bonini, M. Calandra, R. Car, C. Cavazzoni, D. Ceresoli, G. L. Chiarotti, M. Cococcioni, I. Dabo, A. Dal Corso, S. de Gironcoli, S. Fabris, G. Fratesi, R. Gebauer, U. Gerstmann, C. Gougoussis, A. Kokalj, M. Lazzeri, L. Martin-Samos, N. Marzari, F. Mauri, R. Mazzarello, S. Paolini, A. Pasquarello, L. Paulatto, C. Sbraccia, S. Scandolo, G. Sclauzero, A. P. Seitsonen, A. Smogunov, P. Umari, R. M. Wentzcovitch, *J Phys Condens Matter* **2009**, 21, 395502.

[2] P. Giannozzi, O. Andreussi, T. Brumme, O. Bunau, M. Buongiorno Nardelli, M. Calandra, R. Car, C. Cavazzoni, D. Ceresoli, M. Cococcioni, N. Colonna, I. Carnimeo, A. Dal Corso, S. de Gironcoli, P. Delugas, R. A. DiStasio, Jr., A. Ferretti, A. Floris, G. Fratesi, G. Fugallo, R. Gebauer, U. Gerstmann, F. Giustino, T. Gorni, J. Jia, M. Kawamura, H. Y. Ko, A. Kokalj, E. Kucukbenli, M. Lazzeri, M. Marsili, N. Marzari, F. Mauri, N. L. Nguyen, H. V. Nguyen, A. Otero-de-la-Roza, L. Paulatto, S. Ponce, D. Rocca, R. Sabatini, B. Santra, M. Schlipf, A. P. Seitsonen, A. Smogunov, I. Timrov, T. Thonhauser, P. Umari, N. Vast, X. Wu, S. Baroni, *J Phys Condens Matter* **2017**, 29, 465901.

[3] J. P. Perdew, K. Burke, M. Ernzerhof, *Physical Review Letters* **1996**, 77, 3865.

[4] P. E. Blochl, O. Jepsen, O. K. Andersen, *Phys Rev B Condens Matter* **1994**, 49, 16223.

[5] G. Kresse, D. Joubert, *Physical Review B* **1999**, 59, 1758.

[6] X. Hu, X. Chen, X. Li, C. Xu, *Advanced Functional Materials* **2024**, 2316699.

[7] Z. Ma, R. Bai, W. Yu, G. Li, C. Meng, *ACS Applied Nano Materials* **2023**, 7, 1108.

[8] Z. Xu, G. Chen, F. Yang, J. Jang, G. Liu, F. Xiao, Y. Sun, X. Qiu, W. Chen, D. Su, M. Gu, M. Shao, *Electrochimica Acta* **2023**, 458, 142549.

[9] S. Zhou, C. Chen, J. Xia, L. Li, X. Qian, M. Arif, F. Yin, G. Dai, G. He, Q. Chen, H. Chen, *Small* **2023**, 19, 2302464.

[10] X.-G. Wu, R. Wang, F. Ma, X.-L. Liu, D.-L. Jia, H.-C. Yang, Y.-P. Liu, Z.-X. Wang, H.-Z. Zheng, Y.-N. Zhang, J. Hou, J.-J. Huang, S.-L. Peng, *Rare Metals* **2023**, 42, 1526.

[11] T. Li, Z. He, X. Liu, M. Jiang, Q. Liao, R. Ding, S. Liu, C. Zhao, W. Guo, S. Zhang, H. He, *Surfaces and Interfaces* **2022**, 33, 102270.

[12] Y. Tan, Y. Wang, A. Li, Y. Zhang, Y. Zhang, C. Cheng, *Materials Today Energy* **2022**, 29,

101138.

[13] Z.-H. Zhu, B. Yu, W. Sun, S. Chen, Y. Wang, X. Li, L.-P. Lv, *Journal of Power Sources* **2022**, 542, 231583.

[14] L. Li, Y.-J. Chen, H.-R. Xing, N. Li, J.-W. Xia, X.-Y. Qian, H. Xu, W.-Z. Li, F.-X. Yin, G.-Y. He, H.-Q. Chen, *Nano Research* **2022**, 15, 8056.

[15] Y. Ma, H. Fan, C. Wu, M. Zhang, J. Yu, L. Song, K. Li, J. He, *Carbon* **2021**, 185, 526.

[16] B. Q. Li, C. X. Zhao, S. Chen, J. N. Liu, X. Chen, L. Song, Q. Zhang, *Adv Mater* **2019**, 31, 1900592.

[17] K. Tang, C. Yuan, Y. Xiong, H. Hu, M. Wu, *Applied Catalysis B: Environmental* **2020**, 260, 118209.
